# Green-synthesized ZnO nanoparticles from *Capparis aphylla* enhance rice growth and defense responses against *Rhizoctonia solani*

**DOI:** 10.3389/fpls.2026.1849640

**Published:** 2026-07-29

**Authors:** Musrat Ali, Hina Ali, Altaf Ahmed Simair, Muhammad Ibrahim Khaskheli, Jamal-U-Ddin Hajano, Tanveer Alam Khan, Qurban Ali, Muhammad Farooq Hussain Munis

**Affiliations:** 1 Department of Plant Sciences, Faculty of Biological Sciences, Quaid-i-Azam University, Islamabad, Pakistan; 2 Department of Botany, Government College University, Hyderabad, Sindh, Pakistan; 3 Department of Plant Pathology, Faculty of Crop Protection, Sindh Agriculture University, Tandojam, Pakistan; 4 Department of Biology, College of Science, United Arab Emirates University, Al-Ain, Abu Dhabi, United Arab Emirates

**Keywords:** fungi, green nanotechnology, plant defense enzymes, plant extracts, zinc oxide nanoparticles

## Abstract

**Introduction:**

Fungal diseases pose a major threat to global agriculture, reducing crop yield and quality while contributing to environmental and health concerns through excessive use of chemical fungicides. Green nanotechnology utilizing plant extracts has emerged as a promising approach for managing plant diseases. We provide, for the first time, a green nano-fungicide that demonstrates dual action: direct antifungal efficacy against *Rhizoctonia solani*, enhancement of plant development, and upregulation of rice defense enzymes.

**Methods:**

In this study, zinc oxide nanoparticles (ZnONPs) were synthesized using leaf extract of the indigenous medicinal plant *Capparis aphyla* and characterized for their physicochemical and biological properties. FTIR analysis confirmed the presence of functional biomolecules such as alcohols, thiols, isothiocyanates, and aromatics acting as stabilizing and reducing agents. XRD revealed the crystalline nature of ZnONPs with an average particle size of 21 nm, while EDX and SEM confirmed their elemental composition and morphology.

**Result and discussion:**

The green-synthesized ZnONPs exhibited strong antifungal activity against major phytopathogens, showing the highest inhibition against *Rhizoctonia solani* (85.2%), followed by *Fusarium oxysporum* (75.0%) and *Alternaria alternata* (71.6%) at 1000 µg/mL. SEM and TEM analyses demonstrated severe morphological alterations and membrane disruption in *R. solani* hyphae, suggesting oxidative stress as a key mechanism of action. In greenhouse trials, ZnONP treatment significantly improved rice growth parameters, including biomass, root and shoot length, and dry weight, in a concentration-dependent manner. Furthermore, enhanced activities of SOD, POD, CAT, and proline, along with reduced MDA levels and disease index, indicated improved oxidative defense. RT-qPCR analysis further confirmed upregulation of defense-related genes (*SOD, PR1, PR1a*, and *PPO*). These results demonstrate that *C. aphyla*-mediated ZnONPs offer an eco-friendly, cost-effective, and potent strategy for sustainable crop protection.

## Introduction

The growing global population intensifies challenges for agriculture, including declining crop yields due to biotic and abiotic stresses, changing climate patterns, and limited water resources (Wahab et al., 2024). Among these, fungal diseases are a major threat to agriculture, human health, and the environment (Rameez et al., 2024). Pathogenic fungi from Ascomycetes (e.g., *Verticillium*, *Alternaria*, *Fusarium*) and Basidiomycetes (e.g., *Rhizoctonia*, *Sclerotium*) can impair both plant growth and yield, causing significant economic losses (Shuping and Eloff, 2017). Notably, pathogens such as *Rhizoctonia solani*, *Fusarium* spp., and *Phytophthora* spp. can infect roots, stems, leaves, and fruits of over 200 plant cultivars, contributing to considerable pre- and post-harvest losses (Ali et al., 2022). Developing sustainable disease management strategies is crucial to reducing reliance on chemical fungicides. While pesticides have historically enhanced crop yields (Hassaan and El Nemr, 2020), excessive use of chemical fungicides raises environmental and health concerns, including toxicity to non-target organisms, development of resistant pathogens, and potential carcinogenic effects (Tozlu et al., 2018). Therefore, innovative, eco-friendly approaches are urgently needed to safeguard crops and ensure sustainable agricultural productivity.

Nanotechnology has recently emerged as a promising approach for managing fungal diseases. Recognized as a major advancement of this century, it enables the development of innovative materials and solutions with broad societal benefits (Mallick and Rahman, 2025). Green nanotechnology, in particular, focuses on synthesizing nanoparticles from sustainable natural resources using environmentally friendly methods, making them safe and eco-conscious (Gangwar and Joseph, 2025). The integration of nanotechnology with green chemistry has thus become central to modern nanoscience (Jandrotia et al., 2024). The exhibited biostatic and biocidal characteristics of diverse nanoparticles against human and animal pathogens contributed to their investigation for the management of phytopathogens (Kaur et al., 2020). Advances in nanoscience have expanded research on nanoparticles, including plant-mediated green synthesis (Alyamani et al., 2021). Nanoscale technologies have the potential to transform agriculture and food systems, giving rise to the field of agronanotechnology. This approach offers effective, sustainable solutions for disease management and advances both scientific and industrial applications (Elumalai et al., 2025). Green nano-fungicides and Nanoparticles (NPs) can mitigate the impact of resilient fungal pathogens and reduce crop losses (Dávila Costa and Romero, 2025). Phytochemicals in plant extracts act as natural reducing and capping agents, influencing the properties and functionality of NPs for diverse applications. Commonly studied NPs for plant disease control include iron, zinc, silver, and various non-metal oxides or alumino-silicates (Nocito et al., 2025).

Plant-mediated synthesis of zinc oxide nanoparticles (ZnONPs) is an eco-friendly approach that utilizes various plant parts, including fruits, leaves, stems, seeds, and roots (Resmi et al., 2021; Ibrahim et al., 2024). Bio-reduction, the process converting metal ions into zero-valent NPs, is driven by phytochemicals such as vitamins, terpenoids, polysaccharides, polyphenols, alkaloids, and amino acids, with proteins containing amino groups (-NH2) playing a key role in zinc ion reduction. ZnONPs exhibit high biocompatibility and broad applications in tissue engineering, regenerative medicine, drug delivery, sunscreen formulations, antimicrobial coatings, and photocatalysis (Mandal et al., 2022).

ZnO nanoparticles dissolve in aqueous environments under acidic conditions (pH< 7), resulting in the release of Zn²^+^ as H^+^ ions consume OH^-^ ions. In standard microenvironments of plant infection, ZnO nanoparticles can liberate significant quantities of Zn2+ within hours (David et al., 2012). Conversely, at neutral to alkaline pH (7–9), the dissolution rate diminishes but does not cease, especially in the presence of organic acids or chelators. Zinc Oxide is widely acknowledged for its safety profile, which has been validated for its nanoscale application under regulated conditions. These particles are typically classified as Generally Recognized as Safe (GRAS) by regulatory bodies owing to their non-toxic characteristics and extensive history of utilization (Rani et al., 2025). Furthermore, the United States Environmental Protection Agency (US-EPA) has permitted zinc oxide as a stabilizer in pesticide formulations, with concentrations not exceeding 15% weight/weight or weight/volume, so endorsing its acknowledged function in agricultural products (Kalia et al., 2020).

*Capparis aphylla*, a small shrub native to the Indian subcontinent, Saudi Arabia, and Africa, is valued for its therapeutic and nutritional properties (Eddouks et al., 2017). Its shoots, along with *Peganum harmala* leaves, are traditionally used as an anti-fertility remedy (Munawar et al., 2023), and its plant parts are consumed for their bioactive compounds and nutritional value (Khalid et al., 2021). Leveraging *C. aphylla* for ZnONP synthesis offers the potential to integrate its phytochemical richness into sustainable agricultural and biomedical applications. Zinc plays vital roles in plants, participating in enzyme function, nitrogen metabolism, protein synthesis, hormone regulation, and antioxidant defense (Chen et al., 2024). ZnONPs have emerged as an effective zinc source and biostimulant, enhancing nutrient uptake, photosynthetic efficiency, antioxidant capacity, and overall plant growth (Chen et al., 2024). ZnONPs also improve crop quality, increase zinc content in grains, and confer resistance to stresses such as rice blast disease (Qiu et al., 2023). Despite their demonstrated benefits, the commercial availability of ZnONPs varies regionally and is influenced by regulatory and safety considerations (Tolisano and Del Buono, 2023).

This study explores the green synthesis of ZnONPs using the locally available medicinal plant *C. aphylla*. The synthesized nanoparticles were characterized through FTIR, XRD, SEM, and EDX analyses and evaluated as eco-friendly nano-fungicides against rice (*Oryza sativa* L.) fungal pathogen *R. soani*. We hypothesize that green ZnONPs can mitigate fungal stress in rice by enhancing physiological and morphological changes, reducing oxidative stress, and sustaining growth and productivity. The study further investigates the effects of different treatments and concentrations on growth parameters, oxidative stress markers, and physiological responses, aiming to provide sustainable strategies for improving rice resilience and productivity.

## Materials and methods

### Fungal strains and conditions

Fungal pathogens, namely *Alternaria alternata* (MH553296), *Fusarium oxysporum* (JN232136.1), and *Rhizoctonia solani* (HG934415.1), were obtained from our previously isolated, characterized, and preserved stock maintained at the Molecular Plant Pathology Laboratory, Department of Plant Sciences, Quaid-i-Azam University, Islamabad, Pakistan (Ali et al., 2020, 2021, 2022). For this study, these fungal strains were revived on potato dextrose agar (PDA) medium. The cultures were incubated at 28 °C for seven days to ensure optimal growth and active mycelial development before their use in subsequent experiments.

### Preparation of green ZnO nanoparticles

Leaves of the indigenous medicinal plant *Capparis aphylla* L. (locally known as “Kirrar”) were collected from Sindh, Pakistan, washed thoroughly, and shade-dried under controlled conditions. The dried leaves were ground into a fine powder. For extract preparation, 100 g of powder was mixed with 1000 mL of deionized distilled water, shaken at room temperature for 48 h, boiled for 5 min, and incubated in a water bath at 50 °C for 15 min. The mixture was cooled, filtered through muslin cloth, and further passed through sterilized Whatman No. 1 filter paper. The filtrates were labeled and stored at 4 °C for subsequent *in vitro* antifungal assays and for the synthesis of iron and zinc nanoparticles. ZnONPs were synthesized using *C. aphyla* leaf extract following the method of Ali et al. (2022). The extract was mixed with a 1 mM zinc nitrate solution in a 1:2 ratio, heated, and stirred at 80 °C for 2 h. The resulting color change confirmed nanoparticle formation. The mixture was centrifuged at 10,000 rpm for 15 min, washed with distilled water, and dried at ~100 °C for 3–4 h. The dried powder was calcined at 500 °C for 3 h to obtain crystalline ZnONPs. Before antifungal activity assays, the nanoparticles were characterized using various analytical techniques.

### Characterization of green ZnO nanoparticles

To confirm the formation of nanoparticles, various characterization techniques were employed. The crystalline structure of the samples was analyzed using powder X‐ray diffraction (XRD). Fourier-transform infrared spectroscopy (FTIR) was performed to identify and validate the functional groups present. The surface morphology of the synthesized nanoparticles was examined by scanning electron microscopy (SEM), while the elemental composition was determined using energy-dispersive X-ray (EDX) analysis coupled with the SEM detector. SEM (JEM-100XX) was further used to observe the surface morphology and estimate the average particle size, following the protocols described by Shanmuganathan et al. (2018).

### Antifungal activity of ZnONPs

The antifungal activity of ZnONPs was evaluated *in vitro* against three economically important fungal pathogens: *A. alternata*, *F. oxysporum*, and *R. solani*. The pathogens were cultured on PDA at an optimal temperature of 28 °C for seven days. The antifungal potential of ZnONPs was assessed using the food poisoning method. Briefly, PDA media were supplemented with five concentrations of ZnONPs (10, 50, 100, 500, and 1000 µg/mL). A 4 mm mycelial disc of each fungal pathogen was placed at the center of the ZnONPs-amended PDA plates, while PDA plates without ZnONPs served as the positive control. Following seven days of incubation, the inhibition of mycelial growth was quantified and expressed as a percentage using the formula below.


Growth inhibition percentage = (C−T)/C × 100

### Ultrastructural changes of *R. solani* mycelia

Based on *in vitro* screening, *R. solani* was identified as the fungus most effectively suppressed by ZnONPs and was selected for subsequent *in vitro* and *in vivo* experiments. To investigate structural alterations induced by ZnONPs, SEM and transmission electron microscopy (TEM) were employed (Ali et al., 2023). Seven-day-old fungal discs (10 × 10 mm²) were excised from the colony periphery and fixed in 2.5% glutaraldehyde at 4 °C for 24 h. Samples were rinsed three times with phosphate buffer (1 min each) and dehydrated in ethanol for 40 min. Following dehydration, specimens were dried, mounted on aluminum stubs using double-sided carbon tape, and sputter-coated with gold for 1 min before SEM observation at 5 kV using a Hitachi S-3000 N microscope. For TEM analysis, the fixed samples were embedded in Epon 812, ultrathin sections were prepared with an ultramicrotome, and structural details were examined using a Hitachi H-600 TEM.

### Reactive oxygen species assay

The effects of green ZnONPs on *R. solani* mycelium and reactive oxygen species (ROS) production were evaluated using the dichloro-dihydro-fluorescein diacetate DCFH-DA probe and fluorescence microscopy (Ali et al., 2023). *R. solani* samples were treated with ZnONPs and incubated for 12 h at 28 °C. After treatment, mycelia were collected by centrifugation (1200 × g, 8 min, room temperature) and resuspended in 10 mM sodium phosphate buffer (pH 7.4). Samples were then incubated with 10 μM DCFH-DA for 30 min at 30 °C, and fluorescence was observed at 488 nm excitation and 535 nm emission. The experiment was repeated four times for each treatment.

### Pot experiment

The inoculum of *R. solani* was prepared with slight modifications to previously described methods (Ali et al., 2023). Briefly, *R. solani* was first cultured on PDA plates at 28 °C for seven days to obtain well-developed mycelial growth. Sterilized sorghum grains were then inoculated with 1 cm² mycelial plugs cut from the actively growing culture and incubated for two weeks until the grains were completely colonized. The colonized grains were subsequently mixed with sterilized soil at a rate of 10–20 g of inoculum per kilogram of soil. For the control treatment, 100 mL of sterile distilled water was applied to the soil to represent non-infested (healthy) conditions. The inoculated pots were covered with plastic film and incubated at 28 °C for one week to facilitate pathogen establishment. The final inoculum density of *R. solani* in the infested soil was approximately 10^8^ CFU g^-^¹.

Sterilized rice seeds were germinated on filter paper under dark conditions. After one week, seedlings were transplanted into plastic pots (13 × 11 cm²) at a density of 10 seedlings per pot. Eight treatments were applied: Control (only water CK), Control (only *R. solani*), T1 (100 µg/mL ZnONPs), T2 (500 µg/mL ZnONPs), and T3 (1000 µg/mL ZnONPs), T4 (100 µg/mL ZnONPs with *R. solani*), T5 (500 µg/mL ZnONPs with *R. solani*), and T6 (1000 µg/mL ZnONPs with *R. solani*), each in triplicate. ZnONP solutions were stirred for 5 min and sonicated for 30 min to improve solubility. The above-mentioned concentrations of ZnONPs were applied in the drenching soil method (soil properties filed with sandy loam soil of 0.3 kg/pot, pH 5.8, cation 8.5 cmol^+/^kg) near plant roots, four times at regular intervals (Ahmed et al., 2022). After 21 days, plants were harvested, roots were cleared of debris, and the samples were thoroughly washed with double-distilled water. Physiological and biochemical parameters, including shoot and root length, fresh and dry weight, and proline levels, were then assessed following standard protocols (Shahbaz et al., 2023). Overall pot experiment was conducted in growth chamber with controlled parameters.

### Superoxide dismutase activity

Twenty-one days after inoculation, plant leaves were collected following treatment with ZnONPs. The sterile ddH_2_O served as the control (CK). Superoxide dismutase (SOD) activity was determined following Giannopolites and Ries (1977). Fresh leaves were homogenized in 4 mL of phosphate buffer (pH 7.0) containing 1% PVP and 0.2 mM ascorbic acid, then centrifuged at 15,000 × g for 30 min. The supernatant was collected as the enzyme extract. For the assay, 1 mL of extract was combined with 0.1 mM EDTA, 2 mM riboflavin, 75 µM NBT, 13 mM methionine, and 50 mM phosphate buffer (pH 7.8). The homogenate were centrifuged at 12,000 rpm for 20 mints at 4°C, the supernant was collected. Absorbance was measured at 560 nm, and one unit of SOD activity was defined as the amount of enzyme required to inhibit NBT reduction by 50%.

### Peroxidase activity

Peroxidase (POD) activity was determined by measuring the increase in absorbance at 470 nm during guaiacol oxidation. The reaction mixture contained 25 µL of enzyme extract, 0.08% guaiacol, 0.03% H_2_O_2_, and phosphate buffer. The homogenate was centrifuged at 12,000 rpm for 20 minutes, and at 4°C, the supernatant was collected. Absorbance was recorded at 470 nm, and one unit of enzyme activity was defined as an increase of 1.0 in absorbance under these conditions (He and Huang, 2010).

### Catalase and malondialdehyde activity

Catalase (CAT) activity was assayed by measuring the decrease in absorbance at 240 nm over 1 minute due to H_2_O_2_ decomposition, following He and Huang (2010). The reaction mixture contained 50 mM phosphate buffer (pH 7.4) and 15 mM H_2_O_2_. One unit of catalase was defined as the amount of enzyme causing a decrease of 1 in absorbance at 240 nm. Malondialdehyde (MDA) content was determined using the thiobarbituric acid (TBA) method as described by Zhang and Qu (2003).

### Expression profiling of genes related to stress tolerance

Rice plant samples were collected twenty-one days after ZnONPs exposure. Total RNA was extracted using TRIzol reagent (Invitrogen Biotech, USA), and cDNA was synthesized with reverse transcriptase (Takara Bio Inc., Tokyo, Japan). Primers for stress-related genes (*SOD, PR1, PR1a*, and *PPO*) were designed via Integrated DNA Technologies’ Primer Quest tool based on NCBI GenBank sequences (**Supplementary Table 1**), with actin as the reference gene. RT-qPCR was performed on a QuantStudio RT Thermocycler (Thermo Fisher Scientific, USA) using ChamQ SYBR Green Master Mix (Vazyme, China) under the following conditions: 95 °C for 30s, then 40 cycles of 95 °C for 10s and 60 °C for 30s. Relative gene expression was calculated using Ct values normalized to actin (Sikand et al., 2012).

### Assessment of disease incidence

At the end of the experiment, both disease incidence and plant growth promotion parameters were evaluated using a 0–5 disease rating scale for leaf sheaths and leaf blades, adapted from a previous method (Ali et al., 2025). The scale was defined as follows: 0 = no visible symptoms, 1 = appearance of water-soaked lesions, 3 = development of necrotic lesions, 4 = necrosis affecting less than 50% of the leaf area, and 5 = complete necrosis of the leaf leading to leaf death. no stem or root symptoms attributable to *R. solani* were detected in any of the surveyed fields.

The following formulas were used to calculate disease incidence (DI), disease severity index (DSI), and control efficacy (CE):


$$\text{DSI}\left(\%\right)=\frac{\sum \left(\text{Number of diseased plants}\times \text{Disease rating scale}\right)}{\text{Total number of plants assessed}\times \text{Maximum disease rating scale}}\times 100$$

$$\text{DSI}\left(\%\right)=\frac{\text{Number of diseased plants}}{\text{Total number of plants assessed}}\times 100$$

$$\text{CE}\left(\%\right)=\frac{\text{DSI of control}–\text{DSI of treatment}}{\text{DSI of control}}\times 100$$

### Statistical analysis

The analysis of variance was performed using IBM SPSS Version 20.0 (SPSS Inc., Chicago, IL, USA). Duncan’s multiple range test was used to determine significant differences among treatments at p-values< 0.05. Graphical representations were created using Origin’s graphics and analysis program (version 2023, Origin Lab Corporation). All figures were edited, merged, and updated with Adobe Illustrator 2022.

## Results

### Characterization of green ZnONPs

**Figure 1:**
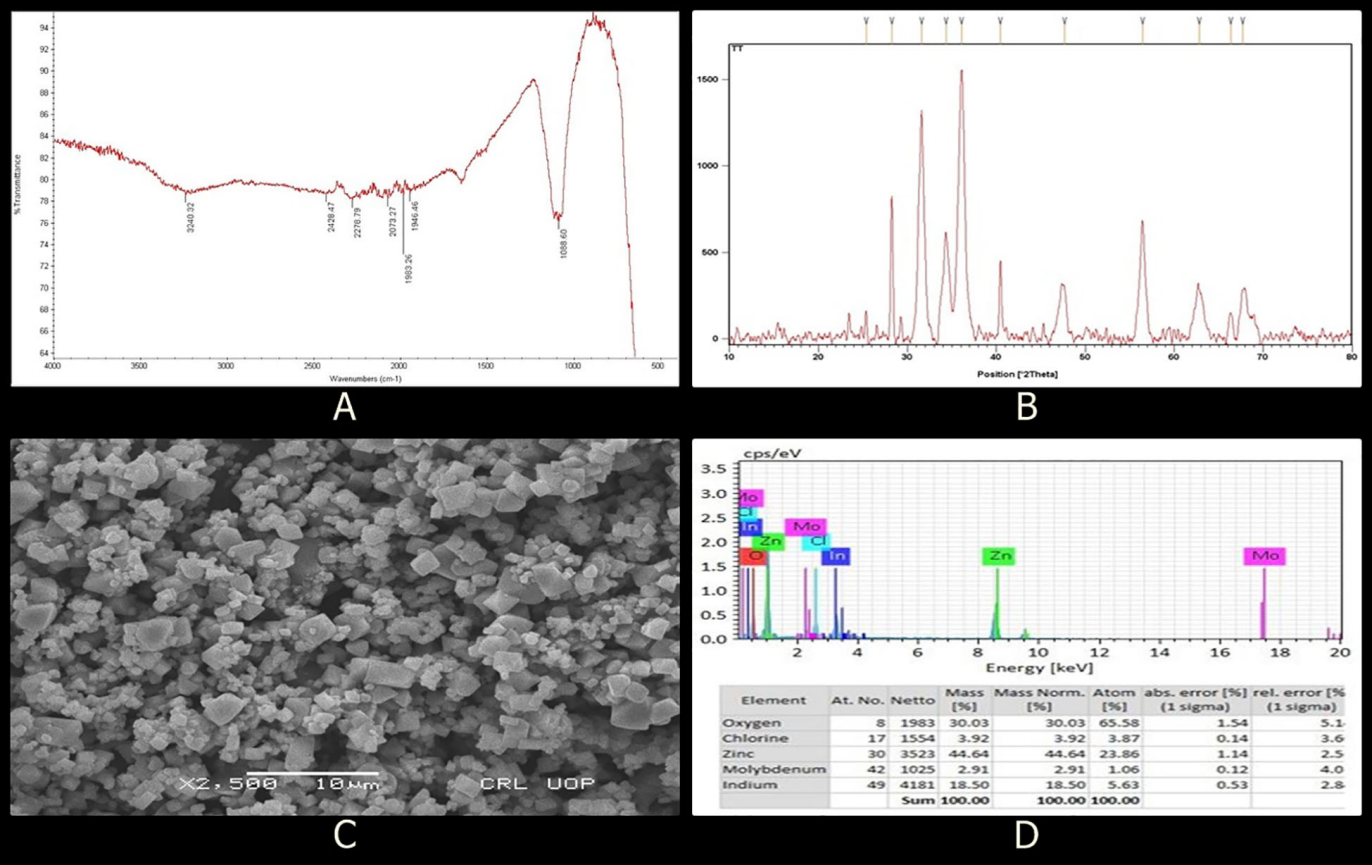
Characterization of green ZnO nanoparticles synthesized using *C. aphyla*. **(A)** FT-IR spectrum of green ZnO nanoparticles prepared with *C. aphyla* aqueous extract, showing functional group peaks responsible for nanoparticle reduction. **(B)** XRD diffraction pattern indicating the average particle size. **(C)** SEM image showing the morphology of the nanoparticles. **(D)** EDX spectrum confirming the elemental composition of the synthesized nanoparticles.

FTIR analysis of the synthesized ZnONPs was performed in the range of 500–4000 cm^-^¹ to elucidate their chemical composition (**Figure 1A**). A prominent peak at 3240.32 cm^-^¹ corresponds to O–H stretching, indicative of alcohol groups and Zn–O vibrations. Peaks at 2428.47 cm^-^¹ and 2073.27 cm^-^¹ were assigned to S–H (thiol) and N=C=S (isothiocyanate) stretching, respectively, while 2278.79 cm^-^¹ represents O=C=O stretching of carbon dioxide. Additional peaks at 1993.26, 1946.46, and 1088.60 cm^-^¹ correspond to allene, aromatic, and secondary alcohol groups. These functional group signals confirm the successful green synthesis of ZnONPs.

Powder XRD was used to characterize the crystalline structure and estimate the crystallite size of the phyto-fabricated ZnONPs. The XRD pattern of the synthesized NPs was recorded over a 2θ range of 10°–80°, confirming the successful conversion of zinc nitrate hexahydrate into ZnO via calcination (**Figure 1B**). The pattern exhibited sharp, well-defined peaks at 2θ values of 25.37°, 28.24°, 31.60°, 34.34°, 36.12°, 40.46°, 47.71°, 56.45°, 62.82°, 66.38°, and 67.73°, corresponding to the (210), (211), (100), (002), (101), (331), (102), (110), (103), (200), and (112) crystal planes, respectively. These peaks indicate a single-phase hexagonal wurtzite structure (P6_3_mc, space group 186; JCPDS card no. 01-079-0207). The average crystallite size, calculated using the Debye–Scherrer equation, was ~21 nm.

The surface morphology of green-fabricated ZnONPs was characterized using SEM (**Figure 1C**). ZnONPs synthesized with *C. aphyla* leaf extract showed predominantly hexagonal-like structures with some agglomeration into clusters, and particle sizes were in the nanometer range. The EDX spectrum (**Figure 1D**) revealed Zn and O weight ratios of 44.64% and 30.03%, respectively, consistent with theoretical values and indicating high purity of the synthesized ZnONPs.

### Antifungal activity of green ZnONPs against fungal pathogens

**Figure 2:**
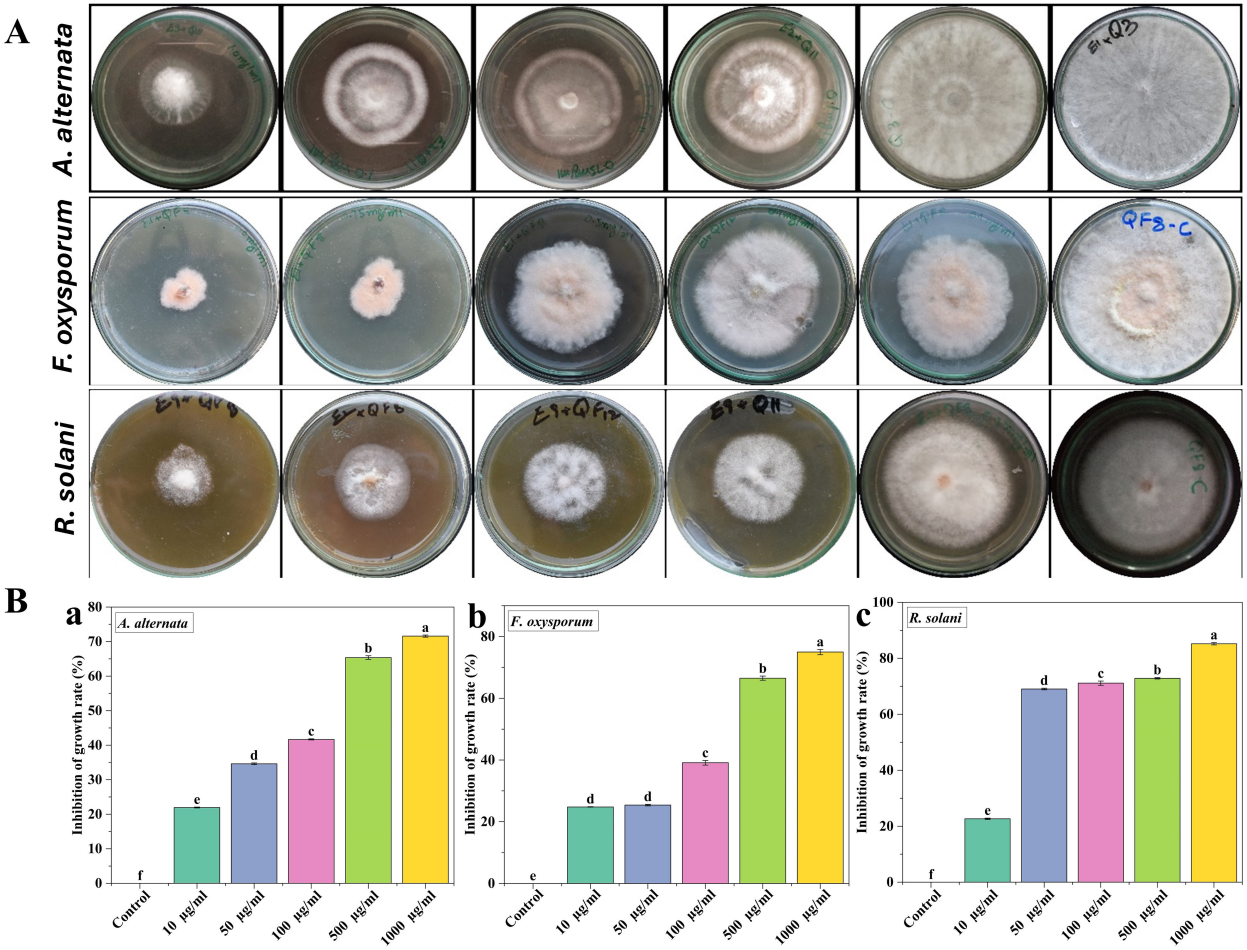
Broad-spectrum activity of ZnONPs against fungal pathogens. **(A)** Visual representation of the growth inhibition (%) of *A. alternata*, *F. oxysporum*, and *R. solani*. **(B)** Dose-dependent growth inhibition (%) of *A. alternata* **(a)**, *F. oxysporum* **(b)**, and *R. solani* **(c)** treated with green-synthesized ZnONPs. Lowercase letters above the columns indicate significant differences among treatments, as determined by the LSD test at *P* ≤ 0.05.

The findings of this study demonstrate the strong antifungal potential of green ZnONPs against phytopathogenic fungi. Significant growth inhibition was observed across all tested concentrations (**Figure 2**), with the maximum effect at 1000 µg/mL. Mycelial growth was consistently reduced in the treated plates (**Figure 2A**), although the degree of inhibition varied with concentration. At 1000 µg/mL, green ZnONPs significantly inhibited the growth of *R. solani* by (85.2%), *F. oxysporum* by (74.9%), and *A. alternata* by (71.6%) (**Figure 2B**). The overall results collectively indicate that the green ZnONPs possess strong potential as an anti-fungal agent against broad-spectrum fungal pathogens.

### Ultrastructural changes and ROS accumulation of *R. solani*

**Figure 3:**
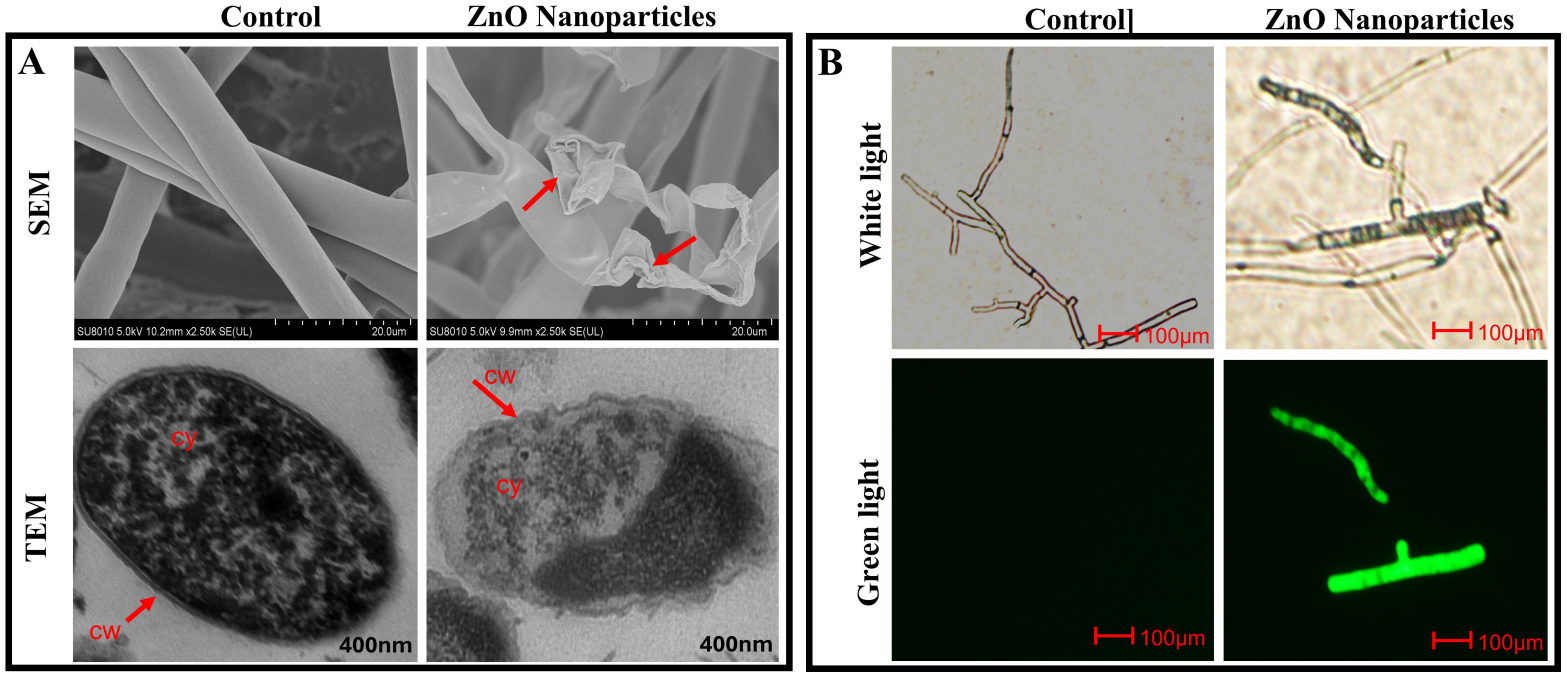
Ultrastructural changes in *R. solani* mycelia induced by ZnONPs observed through SEM and TEM analyses. **(A)** Morphological alterations in *R. solani* mycelia were examined after exposure to ZnONPs using scanning electron microscopy (SEM) and transmission electron microscopy (TEM). The scale bar represents SEM 20μm and TEM 400 nm. **(B)** ROS accumulation in *R. solani* mycelia treated with ZnONPs, visualized under white and green field microscopy using dichloro-dihydrofluorescein diacetate (DCFH-DA) staining. Abbreviations: cy; cytoplasm; cw; cell wall.

The SEM and TEM analyses revealed significant morphological alterations in *R. solani* mycelia upon exposure to ZnONPs. SEM images showed deformed and damaged hyphae in treated samples, whereas control hyphae were thick and uniform (**Figure 3A**). TEM observations indicated that untreated mycelia had intact cell walls, well-defined membranes, and evenly distributed cytoplasm. In contrast, ZnONP-treated mycelia displayed severe ultrastructural disruptions, including cytoplasmic coagulation, organelle disintegration, and cell wall lysis (**Figure 3A**). Green-synthesized ZnONPs induced intracellular ROS production in *R. solani* mycelium, as evidenced by bright green DCFH-DA fluorescence (**Figure 3B**). This indicates oxidative stress in treated mycelium, which disrupts cellular functions, whereas ROS levels in control mycelium remained normal. Fluorescence microscopy revealed morphological changes and increased membrane permeability in treated cells, suggesting reduced viability. These results align with SEM and TEM observations, showing that ZnONPs cause irreversible membrane damage and negatively affect lipids, proteins, and nucleic acids, highlighting their strong cytotoxic effect on *R. solani*.

### Effects of ZnONPs on plant growth traits

**Figure 4:**
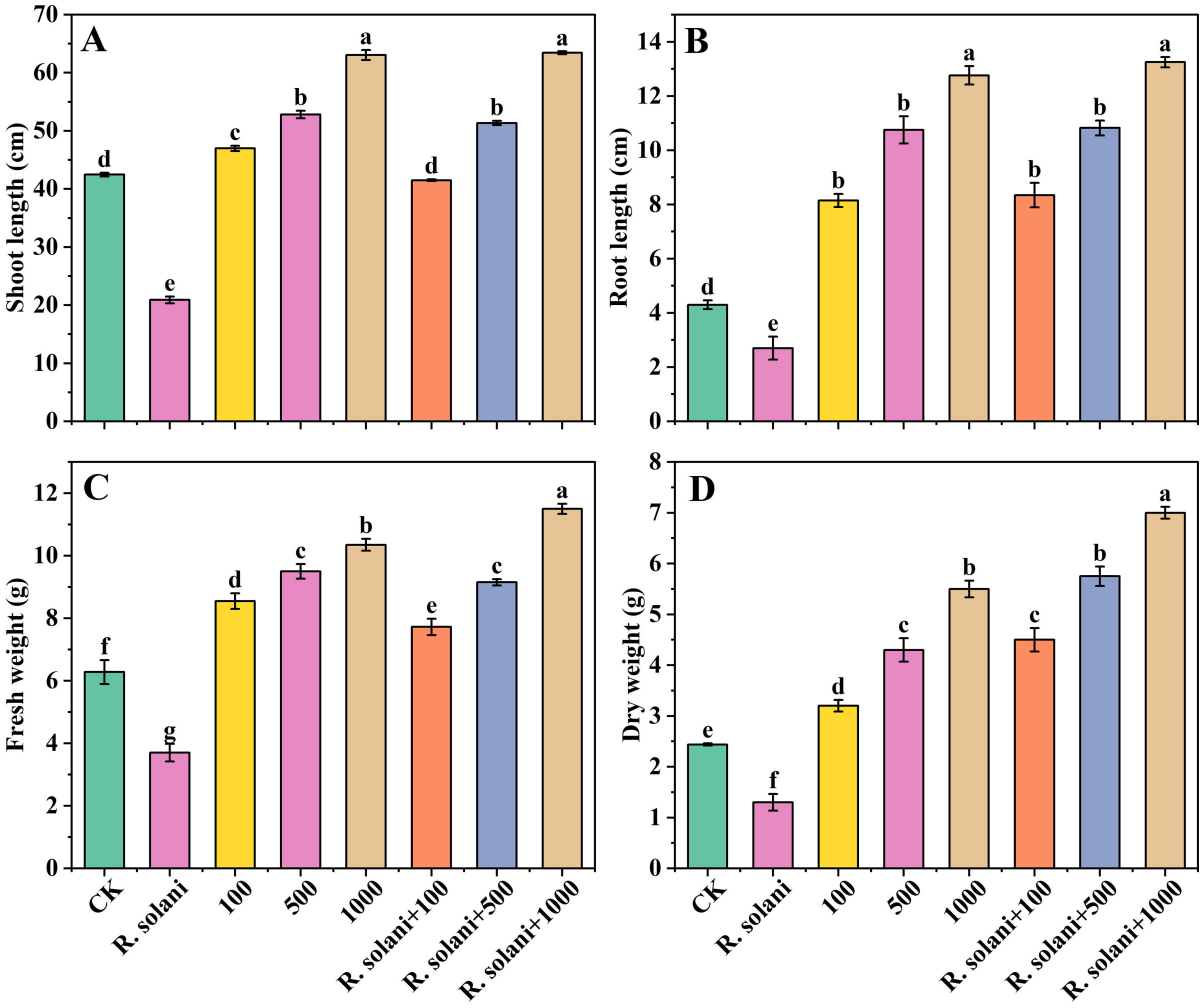
Effect of ZnONPs on rice plant growth promotion in the presence and absence of *R. solani*. **(A)** shoot length, **(B)** root length, **(C)** fresh weight, and **(D)** dry weight. Different lowercase letters above the bars indicate significant differences among treatments, as determined by the LSD test at *P* ≤ 0.05.

To assess the effects of ZnONPs on rice growth and biomass accumulation, we measured shoot and root lengths, as well as fresh and dry weights. In the absence of *R. solani* inoculation, treatment with green ZnONPs significantly enhanced all growth parameters compared with the control, with the most pronounced effects observed at the highest concentration (**Figure 4**). Shoot length (cm) increased by 46.9%, 52.8%, and 63.0% at 100, 500, and 1000 µg/ml, respectively, relative to CK (62.4%) and *R. solani* (20.9%). In *R. solani*-inoculated plants, a similar pattern was observed, where the application of green ZnONPs significantly improved shoot length (cm) by 41.5%, 45.3%, and 63.4% in *R. solani* + 100, *R. solani* + 500, and *R. solani* + 1000 µg/ml treatments, respectively. Green ZnONPs also positively influenced root growth under both inoculated and non-inoculated conditions. In the control (without *R. solani*), root length increased by 8.1%, 10.7%, and 12.7% following treatment with 100, 500, and 1000 µg/ml ZnONPs, respectively. Under *R. solani* inoculation, root length (cm) exhibited slight increases of 8.3% (*R. solani* + 100 µg/ml), 10.8% (*R. solani* + 500 µg/ml), and 13.2% (*R. solani* + 1000 µg/ml) (**Figures 4A, B**). A similar trend was recorded for fresh weight (g), which increased by 8.6%, 9.5%, and 10.4% in the absence of *R. solani* at 100, 500, and 1000 µg/ml, respectively. In inoculated plants, the greatest increase in fresh weight (g) was observed at *R. solani* + 1000 (11.5%), followed by *R. solani* + 500 (9.2%) and *R. solani* + 100 (7.7%), compared with CK (6.3%) and *R. solani* (3.7%). The dry weight (g) also exhibited a consistent increase under both treatments. Under *R. solani* stress, *R. solani* + 1000 µg/ml showed the highest dry weight increase (7.0%), followed by *R. solani* + 500 (5.8%) and *R. solani* + 100 (4.5%). In the absence of *R. solani*, dry weight increased by 3.2%, 4.3%, and 5.5% with 100, 500, and 1000 µg/ml ZnONPs, respectively (**Figures 4C, D**). Collectively, these findings demonstrate that green ZnONPs significantly enhance rice growth and biomass accumulation under both normal and *R. solani*-infected conditions.

### Impact of ZnONPs on antioxidants in rice plants under fungal stress conditions

**Figure 5:**
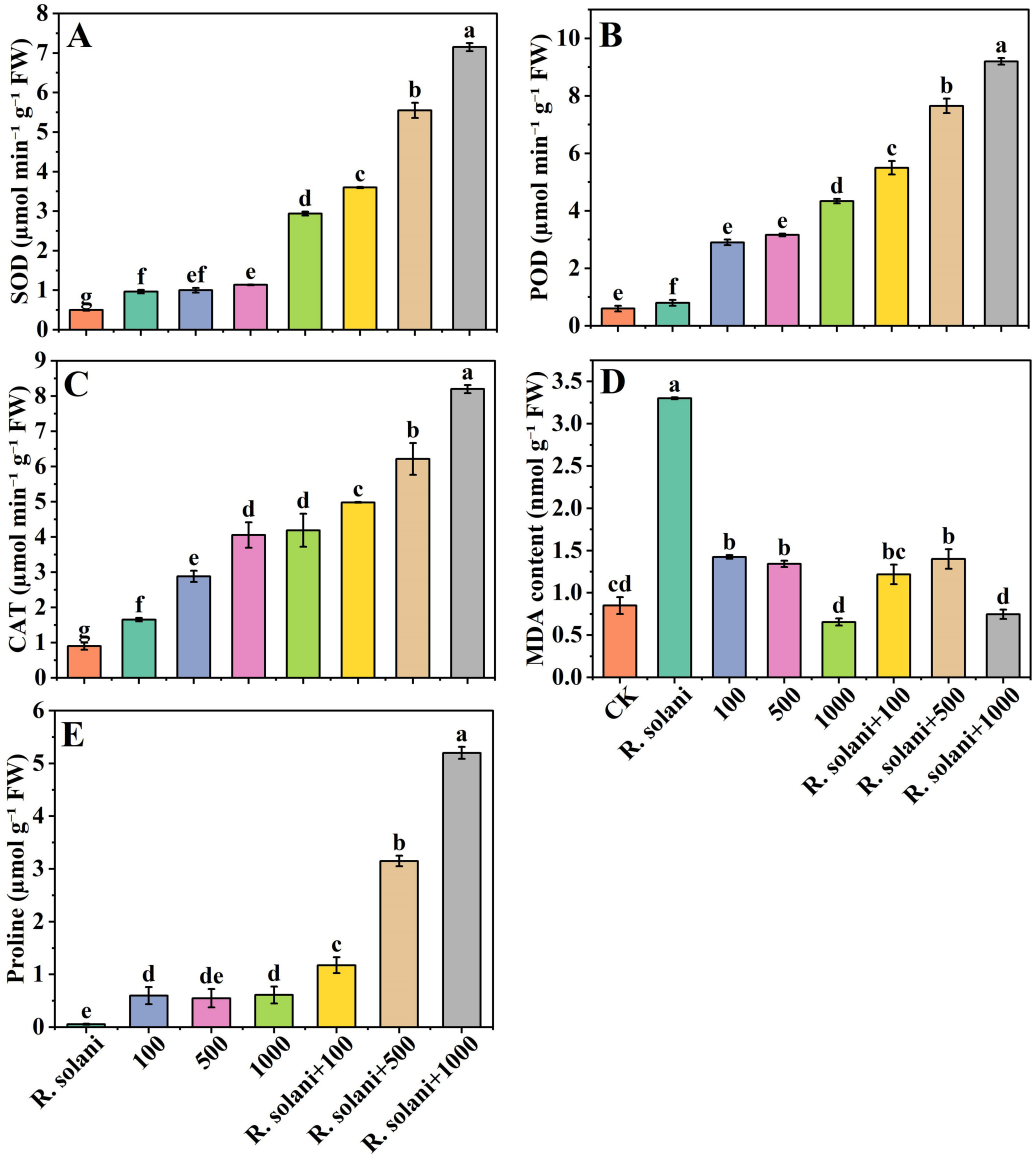
Activities of plant defense enzymes in rice plants treated with different concentrations of ZnONPs in the presence and absence of *R. solani*. **(A)** SOD, **(B)** POD, **(C)** CAT, and **(D)** MDA, **(E)** Proline contents. Different lowercase letters above the bars indicate significant differences among treatments, as determined by the LSD test at *P* ≤ 0.05.

To elucidate the role of ZnONPs in mediating the rice defense response against *R. solani*, the activities of key antioxidant enzymes were quantified in both control and inoculated plants. In non-inoculated rice, the activities of SOD, POD, and CAT increased progressively with higher ZnONP concentrations, reaching maximum levels at 1000 µg/ml. Upon fungal inoculation, these enzymatic activities were further enhanced, with CK + 1000 µg/ml exhibiting the highest activity, followed by CK + 500 µg/ml and CK + 100 µg/ml (**Figures 5A–C**). Lipid peroxidation, assessed by MDA content, was elevated under *R. solani* infection, indicating oxidative stress. However, ZnONP application significantly reduced MDA accumulation, with the lowest levels recorded in CK + 1000 µg/ml (**Figure 5D**). Similarly, proline accumulation increased in response to fungal infection, reflecting enhanced osmotic adjustment, although differences among ZnONP concentrations were relatively minor (**Figure 5E**).

### Relative expression of stress-related genes

**Figure 6:**
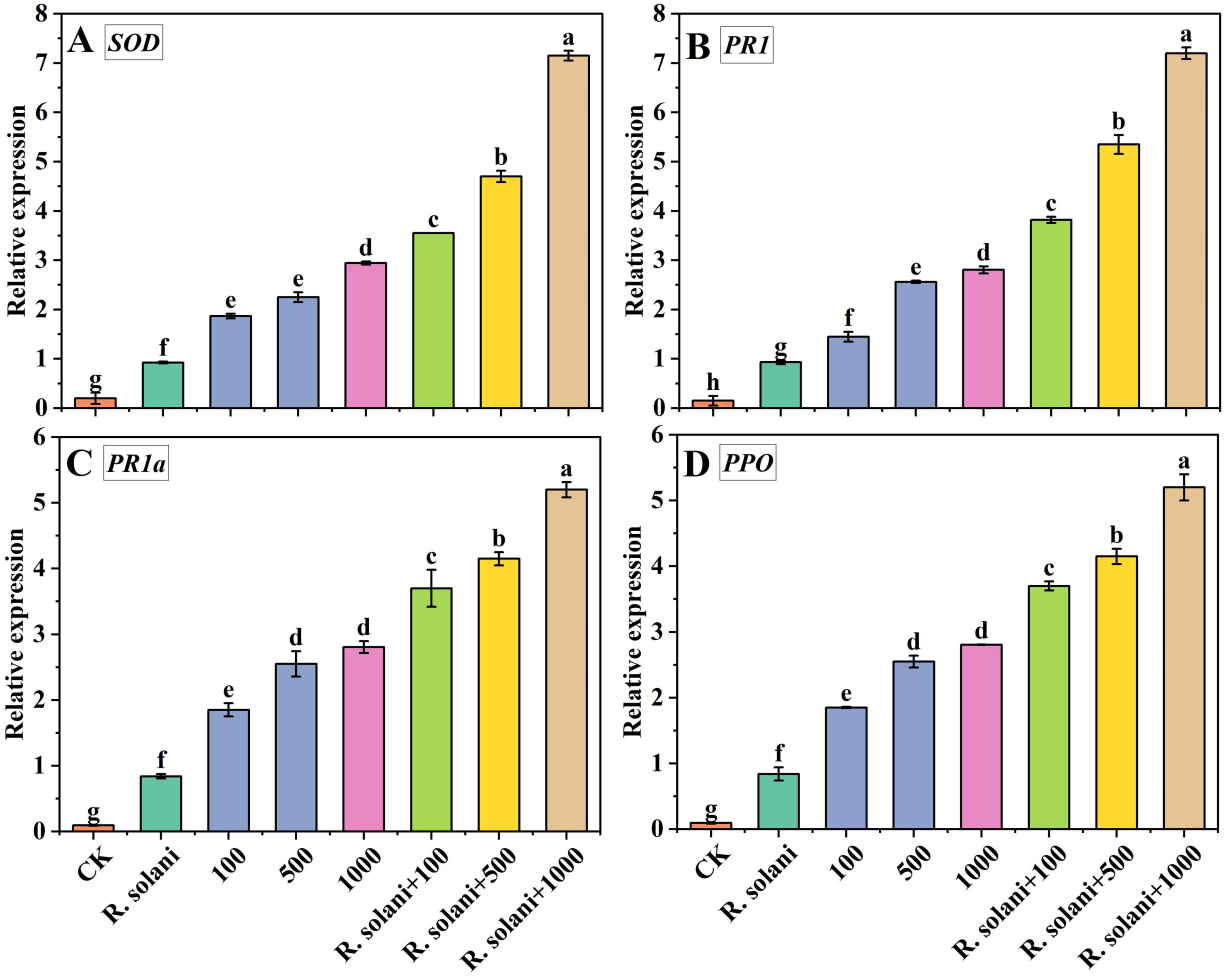
Showing the relative gene expression of **(A)** *SOD*, **(B)** *PR1*, **(C)** *PR1a*, and **(D)** *PPO* in rice under *R. solani* stress conditions without and with ZnONPs concentrations. Different lowercase letters above the bars indicate significant differences among treatments, as determined by the LSD test at *P* ≤ 0.05.

To gain further insights into the role of ZnONPs in enhancing disease resistance in rice, the relative expression levels of defense-related genes (*SOD, PR1, PR1a*, and *PPO*) were analyzed under *R. solani*-inoculated and non-inoculated conditions (**Figure 6**). Overall, plants exposed to fungal stress exhibited a marked upregulation of these genes compared to the CK, while ZnONP treatments further amplified their expression under both stress and non-stress conditions. In the case of *SOD*, gene expression increased by approximately 3-fold under *R. solani*-inoculated conditions, with the highest expression observed in *R. solani* + 1000 µg/mL (3-fold), followed by *R. solani* + 500 µg/mL (2-fold) and *R. solani* + 100 µg/mL (2-fold), compared to CK. Without *R. solani* inoculation, SOD expression was also elevated, showing 1–2 fold increases at 1000, 500, and 100 µg/mL ZnONPs treatments, respectively (**Figure 6A**). Similarly, *PR1* expression exhibited 1-3-fold upregulation under fungal infection, reaching a maximum in *R. solani* + 1000 µg/mL (3-fold), followed by *R. solani* + 500 µg/mL (2-fold) and *R. solani* + 100 µg/mL (1-fold). In non-inoculated rice, ZnONPs treatment resulted in 1–2 fold increases in *PR1* expression relative to the control (**Figure 6B**). For *PR1a*, expression levels showed a 4–5 fold enhancement in *R. solani* + 1000 µg/mL, followed by 1-3-fold in *R. solani* + 500 µg/mL and *R. solani* + 100 µg/mL under *R. solani*-inoculated conditions. Non-inoculated plants also displayed upregulation by 2–3 fold with increasing ZnONPs concentrations compared to CK (**Figure 6C**). The *PPO* gene exhibited a similar expression trend, with *R. solani* + 1000 µg/mL showing the highest increase (3-fold), followed by *R. solani* + 500 µg/mL (2-fold) and *R. solani* + 100 µg/mL (1-fold) under *R. solani* stress. In non-inoculated plants, *PPO* levels were elevated by 2–3 fold with ZnONPs application (**Figure 6D**). Collectively, these results demonstrate that ZnONPs substantially enhance the transcriptional activation of antioxidant and defense-related genes, particularly under pathogen challenge, indicating their potential role in boosting rice defense responses.

### Assessment of disease incidence

**Figure 7:**
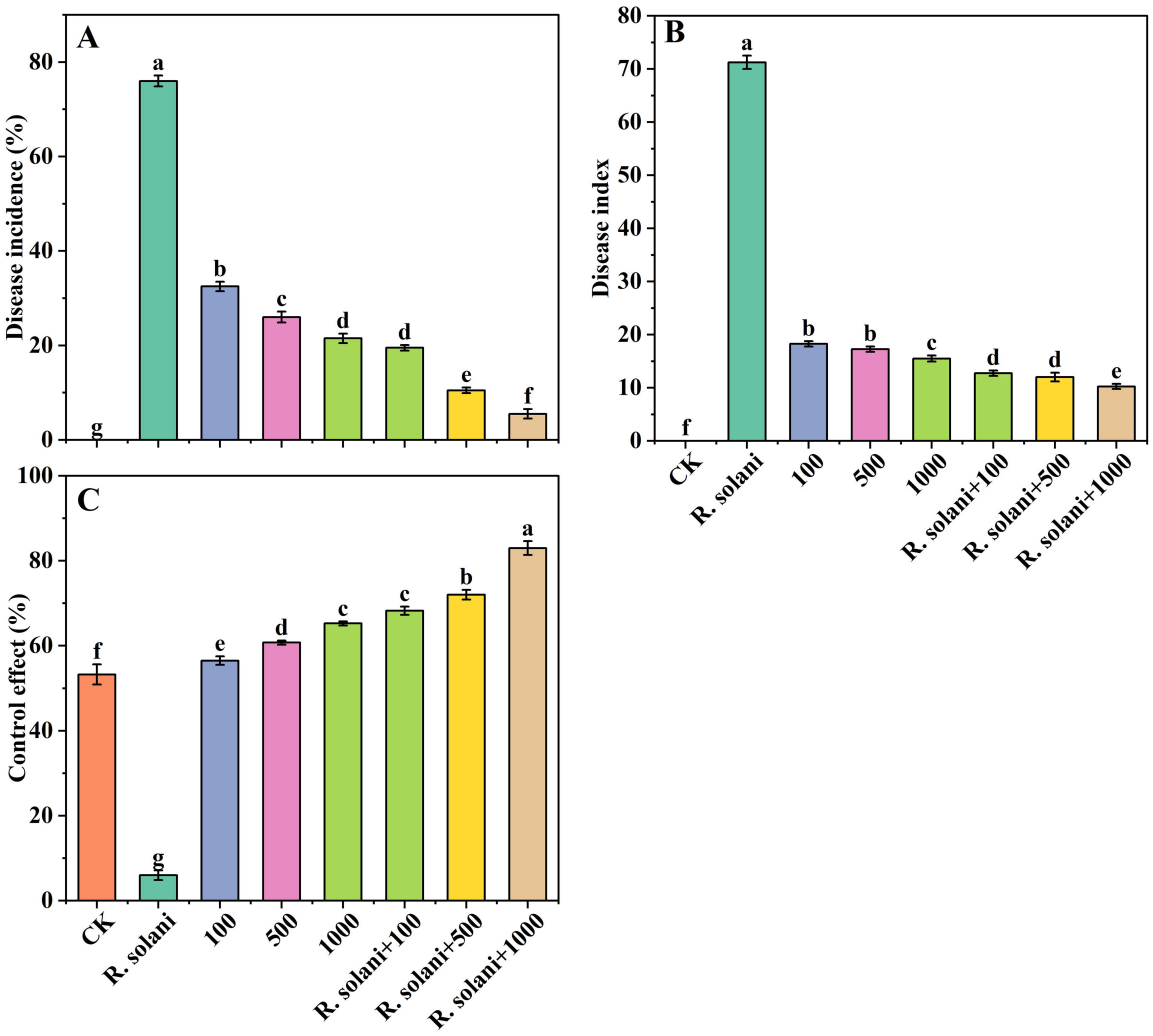
Analyses of the disease index of *R. solani* in the rice plants before and after application of ZnONPs. **(A)** Disease incidence, **(B)** Disease index, **(C)** Control effect. Different lowercase letters above the bars indicate significant differences among treatments, as determined by the LSD test at *P* ≤ 0.05.

To assess the effects of green-synthesized ZnONPs on rice plants under *R. solani* infection and non-infection conditions, disease incidence, disease index, and control efficacy were evaluated. The highest disease incidence (73%) was recorded in plants inoculated with *R. solani* alone, whereas no incidence was observed in CK plants. In the presence of *R. solani*, treatment with 1000 µg/mL ZnONPs resulted in the lowest disease incidence (10%), followed by 500 µg/mL and 100 µg/mL (both 12%). In non-inoculated plants, disease incidence was slightly higher, at 16%, 17%, and 18% for 1000, 500, and 100 µg/mL treatments, respectively (**Figure 7A**). The disease severity index (DSI) also declined with increasing ZnONP concentration in both infected and non-infected plants. Among *R. solani*-infected treatments, DSI values were 5%, 10%, and 19% for 1000, 500, and 100 µg/mL, respectively, compared with 77% in *R. solani* alone. In the absence of infection, DSI values were 23%, 27%, and 33% for 1000, 500, and 100 µg/mL, respectively (**Figure 7B**). Control efficacy was highest in ZnONP-treated plants and lowest in those infected only with *R. solani*. The highest control effect (83%) was observed in the *R. solani* + 1000 µg/mL treatment, followed by 500 µg/mL (72%) and 100 µg/mL (68%). In non-infected plants treated with ZnONPs alone, control efficacy reached 65%, 60%, and 56% for 1000, 500, and 100 µg/mL, respectively. The lowest control efficacy (6%) was recorded in CK plants without ZnONPs treatment (**Figure 7C**).

## Discussion

The management of fungal diseases continues to pose significant challenges for modern phytopathologists (Jeger et al., 2021). With increasing awareness of the environmental and health hazards associated with chemical pesticides, there is a growing shift toward natural and eco-friendly alternatives (Singh, 2024). Recent research has also emphasized the role of metallic oxide nanoparticles, particularly zinc oxide (ZnO), in suppressing phytopathogenic fungi (Daniel et al., 2024). In the present study, green-synthesized ZnONPs were evaluated for their antifungal efficacy against *A. alternata, F. oxysporum*, and *R. solani*. The use of green synthesis offers distinct advantages over conventional chemical and physical methods, including reduced toxicity, lower cost, and environmental sustainability (Corciovă et al., 2022). Characterization of the synthesized nanoparticles confirmed their crystalline and morphological properties (Sankarganesh et al., 2025). XRD analysis indicated an average crystallite size of approximately 21 nm, while SEM images revealed a predominantly cubical morphology. The EDX spectrum confirmed the presence of zinc and oxygen as the major elements, with characteristic peaks at 1.24 and 0.71 keV corresponding to their respective binding energies (Daniel et al., 2024). Such characterization is critical, as impurities can significantly influence the biological activity and stability of nanoparticles (Dönmez, 2021). The results of this study suggest that the ZnONPs synthesized using *C. aphyla* extract were successfully formed and exhibited a high degree of purity. Moreover, elemental analysis indicated that the bimetallic ZnONPs primarily contained zinc and oxygen, with trace amounts of magnesium possibly incorporated from the plant matrix (Poojari et al., 2025).

When compared to previous studies, the antifungal activity observed in this work aligns with findings reported for other green-synthesized ZnONPs (Elshafie et al., 2023). For instance, ZnONPs synthesized using *Azadirachta indica* and *Moringa oleifera* extracts have shown significant inhibition against *Fusarium* and *Alternaria* species, attributed to the nanoparticles’ ability to generate ROS that disrupt fungal cell walls and membranes (Ewekeye et al., 2023). Similarly, study by Ali et al. (2022) demonstrated that smaller particle sizes and higher surface reactivity enhanced antifungal performance. The present study demonstrated that ZnONPs exhibited strong antifungal activity against *R. solani, A. alternata*, and *F. oxysporum*, with maximum inhibition observed at 1000 µg/mL. The inhibition increased proportionally with concentration, confirming a dose-dependent effect. These findings align with previous studies reporting significant antifungal activity of ZnONPs against a range of phytopathogenic fungi (Kamal et al., 2024; (Farhana et al., 2023; Ali et al., 2022). The antifungal mechanism of ZnONPs is likely linked to their ability to generate ROS and disrupt cell membrane integrity observed in SEM and TEM. A similar result found that mycelium exhibited a healthy morphology characterized by a smooth and turgid surface (Mishra et al., 2025). Additionally, Djearamane et al. (2022) documented morphological alterations in *Candida albicans* using SEM. Metal-based nanoparticles can penetrate fungal cell walls, alter membrane permeability, and interfere with essential biomolecules such as RNA, DNA, and proteins (Kamal et al., 2022). This cellular damage ultimately leads to growth inhibition and cell death. A similar pattern of results was found for the green synthesized ZnONPs, which supports the current findings (Nehru et al., 2023).

This study demonstrated that ZnONPs significantly improved the growth and biomass of rice plants, both in the presence and absence of *R. solani*. Among the tested concentrations, 1000 µg/ml ZnONPs produced the greatest increase in fresh biomass levels compared to the control. These findings suggest that ZnONPs enhance plant metabolism and promote better physiological performance. The improved growth may be attributed to enhanced nutrient uptake, photosynthesis, and auxin regulation stimulated by ZnONPs. Similar results were reported by Abdelaziz et al. (2022), who observed increased shoot and root length, chlorophyll, and protein accumulation in ZnONP-treated plants. The nanoparticles likely improved stomatal conductance and intercellular CO_2_ concentration, enhancing gas exchange and chlorophyll synthesis (Kareem et al., 2022). Furthermore, in this study, ZnONPs also stabilized cell membranes, minimized water loss, and promoted cellular protection, resulting in higher root and shoot biomass (Dabaghkar et al., 2024). Moreover, our investigated study aligns with previous reports showing that ZnONPs suppress various plant pathogens while promoting growth and yield (Mosquera-Sánchez et al., 2020). The increased proline levels in treated plants further indicate a stress-mitigating effect. Proline acts as an osmoprotectant and antioxidant, maintaining redox balance and scavenging reactive oxygen species (Siddiqui et al., 2019). Therefore, the enhanced proline and sugar accumulation observed in this study suggests improved tolerance to oxidative stress.

Abiotic and biotic stresses disrupt the balance between ROS and the antioxidant defense system, leading to oxidative damage and reduced plant vitality (Riaz et al., 2025). In this study, ZnONPs significantly enhanced the antioxidant response in rice under *R. solani* stress. Plants treated with ZnONPs showed higher activities of SOD, CAT, and POD, along with reduced MDA levels, indicating effective mitigation of oxidative stress. ZnONPs acted as cofactors for antioxidant enzymes and directly scavenged ROS, aligning with the findings of Li et al. (2013), who reported that zinc treatment minimized lipid peroxidation and oxidative injury. The ZnONP treatments exhibited the strongest antioxidant defense and lowest oxidative stress markers, suggesting its optimal effectiveness under stress conditions (El Sabagh et al., 2019). Moreover, ZnONPs improved osmotic regulation and protein stability by increasing proline contents, supporting earlier observations in maize (Sun et al., 2020). Zinc also plays a crucial role in maintaining membrane integrity, protein synthesis, and tryptophan formation (Taran et al., 2017). The ability of ZnONPs to enter plant tissues through seeds, roots, or leaves enhances their regulatory effects on stress-related signaling pathways, including calcium ions, nitric oxide, phytohormones, and transcription factors (Singh et al., 2025).

Our findings confirm that ZnONPs enhance plant defense by activating defense-related genes *SOD*, *PR1*, *PR1a*, and *PPO*, improving growth and physiological traits in treated rice compared to controls. Similar studies have shown that ZnONPs trigger SA-dependent and ROS-mediated signaling pathways that strengthen resistance against a broad range of pathogens (Zhao et al., 2022). Nanoparticles influence gene expression through complex transcriptional regulation networks, where both activation and repression play key roles in plant defense responses (Kumari et al., 2024). The upregulation of antioxidant enzymes supports the results that nanoparticles modulate ROS homeostasis to maintain cellular balance and stress tolerance (Zhang et al., 2024). Green-synthesized nanoparticles can both generate and scavenge ROS, establishing a controlled oxidative environment that activates downstream defense mechanisms, including pathogenesis-related (PR) proteins and antioxidant enzymes (Zhao et al., 2022). PR proteins, especially *PR1*, are crucial markers of systemic acquired resistance, and their overexpression enhances plant immunity (Javed et al., 2025). Likewise, polyphenol oxidase (*PPO*) activity contributes to pathogen defense by promoting phenolic oxidation and lignin formation, reinforcing plant cell walls (Fernandes, 2023). Overall, these findings suggest that ZnONPs induce a coordinated defense response involving transcriptional regulation, redox balance, and the activation of key defense proteins. However, variations in nanoparticle synthesis and application highlight the need for standardized experimental protocols to ensure reproducibility across studies (Guo et al., 2023).

## Conclusion

**Figure 8:**
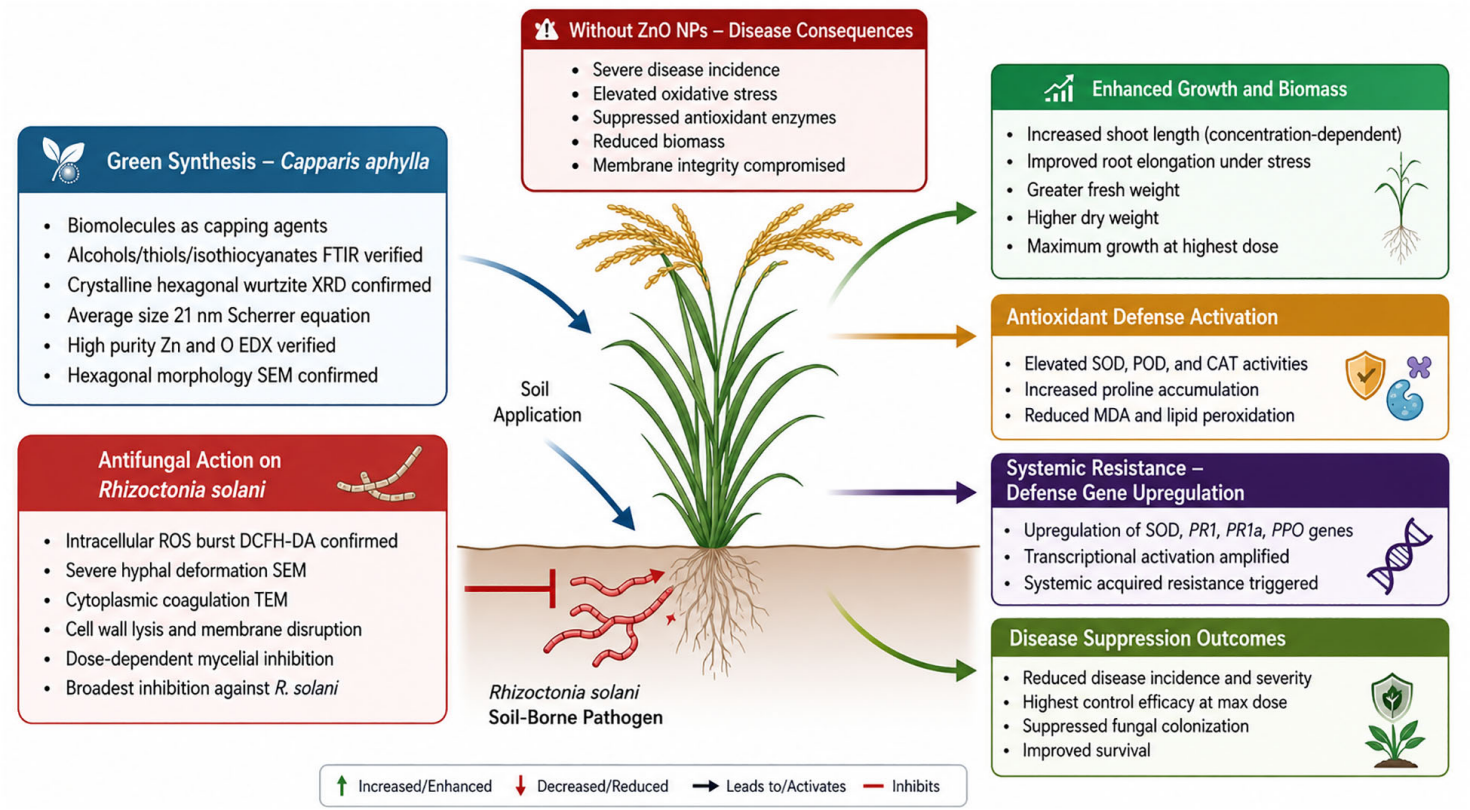
Proposed mechanism of green-synthesized ZnO nanoparticles (ZnONPs) from *Capparis aphylla* in rice. Soil-applied ZnONPs directly inhibit *R. solani*, enhance antioxidant defenses, upregulate defense-related genes (*SOD*, *PR1*, *PR1a*, and *PPO*), promote plant growth, and reduce disease severity, resulting in improved systemic resistance and biomass accumulation.

Green extract of *C. aphyla* can efficiently mediate the synthesis of ZnONPs, which improves physiological, biochemical, and morphological attributes of the rice plant. This improved plant health reduces disease incidence caused by *R. solani.* This is an eco-friendly way of controlling important diseases of the rice plant by avoiding the use of toxic chemical fungicides. Continued research is warranted to establish safe application thresholds and to optimize the use of ZnONPs for sustainable crop production and effective disease management. A proposed model illustrating the direct and indirect mechanisms of ZnONPs in managing rice diseases is presented in **Figure 8**.

## Data Availability

The original contributions presented in the study are included in the article/**Supplementary Material**. Further inquiries can be directed to the corresponding authors.
