# Supplementary material for: Green-synthesized ZnO nanoparticles from Capparis aphylla enhance rice growth and defense responses against Rhizoctonia solani
Source: Front Plant Sci. 2026 Jul 29;17:1849640. doi: 10.3389/fpls.2026.1849640 (PMC13461608; doi:10.3389/fpls.2026.1849640)
Supplement: Supplementary file 1 [file Table1.docx]

***Supplementary file***

**Table S1:** Primers used for defense-related genes in rice.

| *PR-1* (F) | GGCTAATCTTTCCCAGGCG |
| --- | --- |
| *PR-1* (R) | TTGCAGTCGTTGATCCTCTG |
| *PR1a*-F | TCTTCATCACCTGCAACTACTC |
| *PR1a*-R | ATTCATCGGATTTATTCTCACC |
| *SOD* (F) | TTGGGAATTGATGTCTGGGAG |
| *SOD* (R) | ACAGCGGGAAACTCAAGAG |
| *PPO-*F | CGCCTTACGTTCTTGGGAAT |
| *PPO-*R | TTGAACCACGGACAGTACCA |
| *Actin* (F) | TGGATCGGTGGCTCTATTTTG |
| *Actin* (R) | TTTGTAAGTCCCCTTCACCG |
